# β2-Adrenergic receptor expression in subchondral bone of patients with varus knee osteoarthritis

**DOI:** 10.1515/med-2022-0498

**Published:** 2022-06-07

**Authors:** Xiaochun Yang, Xuegang Liang, Haohui Guo, Long Ma, Li Jian, Xin Zhao, Jian Wang, Lvlin Yang, Zhiqiang Meng, Qunhua Jin

**Affiliations:** Department of Orthopedics Ward 3, The General Hospital of Ningxia Medical University, Yinchuan, 750004, Ningxia, China; Department of The General Hospital of Ningxia Medical University, Ningxia Medical University, Yinchuan, 750004, Ningxia, China; Department of Pathology, The General Hospital of Ningxia Medical University, Yinchuan, 750004, Ningxia, China

**Keywords:** osteoarthritis, β2-adrenergic receptor, mechanical stresses, subchondral bone, bone remodeling

## Abstract

An important causative factor in osteoarthritis (OA) is the abnormal mechanical stress-induced bone remodeling of the subchondral bone. β2-adrenergic receptor (Adrb2) plays a major role in mechanical stresses that induce bone remodeling. The medial tibial plateau (MTP) and lateral tibial plateau (LTP) of patients with varus Knee osteoarthritis (KO) bear different mechanical stresses. The present study aimed to investigate the expression of Adrb2 in medial tibial plateau subchondral bone (MTPSB) and lateral tibial plateau subchondral bone (LTPSB) in patients with varus KO. A total of 30 tibial plateau samples from patients undergoing total knee arthroplasty for varus KO and MTPSB and LTPSB were studied. Statistical analysis was performed using paired sample *t*-tests. Safranin O-Fast Green staining and Micro-computed tomography showed significant differences in the bone structure between MTPSB and LTPSB. Tartrate-resistant acid phosphatase (TRAP)-positive cell density in MTPSB was higher than that in LTPSB. Immunohistochemistry, reverse transcription-quantitative polymerase chain reaction, and Western blot analysis revealed that compared to LTPSB, the levels of Adrb2, tyrosine hydroxylase (TH), and osteocalcin increased significantly in MTPSB. Double-labeling immunofluorescence showed Adrb2 was present in the majority of TRAP-positive multinuclear cells of the MTPSB. The expression of Adrb2 and TH was significantly higher in MTPSB than in LTPSB, confirming the involvement of these molecules in the development of OA.

## 1 Introduction

Osteoarthritis (OA) is the most common degenerative joint disease, affecting about 250 million people worldwide. It mainly affects weight-bearing joints, such as knee and hip, and is characterized by progressive cartilage degeneration and subchondral bone changes [1]. Abnormal subchondral bone remodeling plays a major role in the pathogenesis of OA. Subchondral bone is the mechanical support for articular cartilage and undergoes bone resorption and remodeling in response to changes in mechanical stresses [2].

Mammalian bones exhibit a sympathetic nervous system (SNS) that is regulated by autonomic and sensory nerves in response to mechanical stresses [3,4]. During normal bone remodeling, sympathetic nerves effectuate via catecholamine, a critical sympathetic nerve neurotransmitter norepinephrine that activates the β2-adrenergic receptor (Adrb2), which in turn inhibits osteoblast proliferation and differentiation and promotes osteoclast precursor maturation and bone resorption activity. In this process, tyrosine hydroxylase (TH) acts as the rate-limiting enzyme for norepinephrine biosynthesis [5,6]. In the orthodontic tooth movement (OTM), mechanical stresses induced Adrb2 activation in alveolar bone remodeling [7]. Sympathetic nerve fibers are detected in the subchondral bone of OA and in a rat temporomandibular experimental OA model [8]. Adrb2 antagonists inhibit subchondral bone loss and osteoclast activity, while Adrb2 agonists aggravate these reactions, and Adrb2 plays a critical role in mechanical stress-induced bone remodeling [9].

When normal walking on a healthy knee, the medial compartment experiences 60–80% weight bearing, and the lesions in the medial compartment of the knee are common due to significant stress and knee adductive torque during weight-bearing activities [10,11]. In the early stage of knee osteoarthritis (KO), mechanical stresses cause changes in the subchondral bone, and the progression of OA increases the varus deformity of the knee [12,13]. This phenomenon increases the mechanical stresses through the medial compartment, further aggravating the degree of OA and varus deformity [14]. However, the expression of Adrb2 in the subchondral bone of human KO has not yet been reported.

The present study selected the same patients with KO varus deformity of the medial tibial plateau (MTP) and the lateral tibia plateau (LTP) and conducted a paired comparison of the subchondral bone structure change to assess the differences in Adrb2 expression. Also, Adrb2 with KO was investigated with respect to the underlying mechanism to provide a reference for the treatment of KO.

## 2 Materials and methods

### 2.1 Sample collection

**Figure 1:**
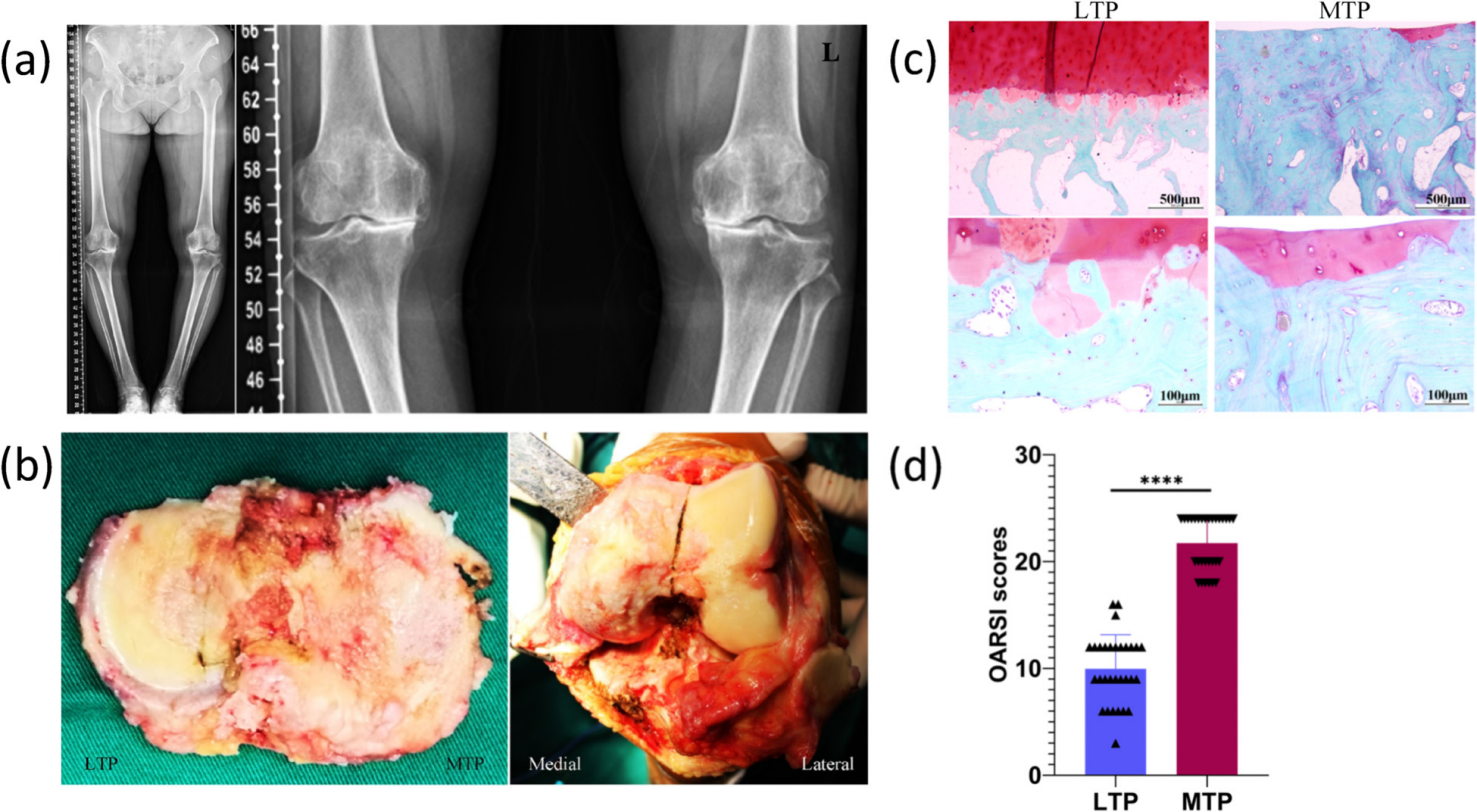
Radiographic, macroscopic observations and histological evaluation of the MTP and LTP. (a) Conventional long-leg radiograph: After standing, the mechanical shaft moves inward, and the stress concentrates on the inside of the knee joint; local magnification of the X-ray film showed that the medial joint space was significantly smaller than the lateral joint space, and the local osteosclerosis on the medial side of the joint was significantly greater than that on the lateral side. (b) Intraoperative gross pathological images: knee medial tibial plateau than the lateral cartilage defect is severe, and some bone is exposed, corresponding to the medial femoral condyle the same performance. The cartilage of the lateral femoral condyle of the knee joint is relatively complete with good gloss. (c) Safranin O/Fast Green staining. (d) OARSI score. Statistical analysis results: The difference between the MTP and LTP groups was statistically significant. Magnification ×50, scale bar 500 µm; magnification ×200, scale bar 100 µm; ^****^ *P* < 0.0001.


Specimens of the tibial plateau were obtained from patients with primary varus deformity KO who underwent total knee arthroplasty in the Department of Orthopedics, Ningxia Medical University General Hospital, Yinchuan, China, from 2019 to 2020. The cohort consisted of 30 cases, including 12 males and 18 females, aged 62–78 (mean age, 66.77 ± 3.48 years) years. The diagnosis of OA was based on the criteria of the American College of Rheumatology [15]. The patients with OA secondary to other diseases, such as trauma and connective tissue disease, were excluded and had no history of β-adrenergic receptor agonist and antagonist drug intake. The preoperative knee X-ray was taken. The measured varus angle was 12.0–25.5° (average: 17.6°). Most of the lesions occurred in the medial compartment (Figure 1a). The severity of OA knee was assessed using Kellgren–Lawrence (K–L) scale (1–4) on weight-bearing radiographs [16]. Of 30 knees, 12 knees were K–L grade 3 and 18 knees were K–L grade 4.

**Ethical approval and informed consent**: This study was approved by the Ethics Committee of Ningxia Medical University General Hospital (No. 2020-985), and informed consent was obtained from each patient prior to inclusion in the study.

### 2.2 Sample processing

The tibial plateau material was obtained during surgery and placed on a clean table. Then, the bone and cartilage in the central bearing area of the MTP (*n* = 30) and LTP (*n* = 30) samples from each patient were selected and trimmed into blocks (2.0 cm^3^ × 2.0 cm^3^ × 1.0 cm^3^). The coronal sections were used to divide the bone and cartilage into two parts randomly. The part of the samples that contained cartilage and subchondral bone was immobilized in 4% paraformaldehyde for 48 h. After micro-computed tomography (CT) analysis, the samples were decalcified with 10% ethylenediaminetetraacetic acid at room temperature for 3 weeks and embedded in paraffin. Safranin O-fast green staining, tartrate-resistant acid phosphatase (TRAP) staining, immunohistochemistry, and immunofluorescence were performed in 4-µm-thick discontinuous sections. The other part removes cartilage, and the subchondral bone was wrapped in foil and stored at −80°C to measure the mRNA and protein levels subsequently.

### 2.3 Safranin O-fast green staining

The processes were described previously [17]. The sections were stained with hematoxylin (ZLI-9610, ZSGB-Bio, Beijing, China) for 3–5 min and washed with tap water for 10 min. The specimens were dyed with 0.3% solid green (F8130, Solarbio, Beijing, China) for 5 min and quickly washed with the weak acid solution for 10–15 s to remove the residual solid green. The slices were added into 1% Safranin staining solution (S8020, Solarbio, Beijing, China), soaked for 5 min, then rinsed three times with distilled water for 5 min, and dehydrated with 95% ethanol and absolute ethanol. Xylene was transparent and sealed with optical resin. The samples were examined using a microscope. The Image-Pro Plus 6.0 image analysis software (Media Cybernetics, Bethesda, MD, USA) was employed to assess the structural parameters of the cartilage and subchondral bone deduced with morphometric methods. The parameters included total articular cartilage (TAC; the perpendicular distance between the cartilage surface and cement line), subchondral bone plate (SCP) thickness (the distance from the cement line to the interface between the SCP and trabecular bone), and trabecular bone area/total area (BA/TA), which are calculated as follows: (trabecular bone area in the region of measurement)/(trabecular bone area + marrow cavity area) × 100.

Each section was evaluated using the Osteoarthritis Research Society International (OARSI) score [18]. The final score was based on the Safranin O/fast green staining results and the OARSI scoring items. The OARIS score was obtained by multiplying the histological grading of cartilage degeneration (in six grades) by the histological staging of cartilage degeneration (in five stages) following Safranin O/fast green staining.

### 2.4 TRAP staining

The paraffin slices were immersed in xylene for 5 min each to remove paraffin and then re-hydrated in an alcohol series and distilled water for 5 min. Tissue sections were pre-incubated at 37°C for 20 min in TRAP buffer (0.1 M acetate buffer pH 5.0 and 50 mM sodium tartrate). The enzyme reactivity was visualized by incubating the sections in TRAP buffer containing 0.1 mg/mL naphthol AS-MX (N-4875, Sigma, USA) and 0.3 mg/mL fast red violet LB salt (F-3381, Sigma, USA) at 37°C for 1 h. The stained sections were rinsed in phosphate-buffered saline (PBS) and counterstained with hematoxylin (ZLI-9610, ZSGB-Bio, Beijing, China). The slices were dehydrated by placing them in 70, 80, 90, and 100% alcohol and xylene for 1 min each, then sealed with neutral glue, and covered with a coverslip. TRAP-positive osteoclasts were counted within 400 µm of the cement line in the osteochondral junction and divided by the length of the subchondral bone to provide an osteoclast density expressed as TRAP-positive cells per mm^2^ [19]. One dark purplish or reddish cell with ≥3 nuclei was counted as one osteoclast.

### 2.5 Micro-CT evaluation

The LTP and MTP samples were observed under a micro-CT scanner (SkyScan 1076, Bruker, Kontich, Belgium) with the following scanning parameters: 18 μm isotropic voxel size, 55 kV voltage, 109 μA current, 200 ms integration time, and 4,000 projections. The microstructure of the subchondral plate and trabecular bone was visible in the two-dimensional (2D) reconstructed images that were converted into discrete binary objects by the global thresholding and binarization procedures combined with image filtering such as despeckling to remove noise. Subsequently, the data were processed for three-dimensional (3D) measurement in SkyScan CTAn software. The surface-rendered 3D models were created for visualization using the SkyScan CTVol software [20]. For subchondral trabecular bone, a cubic region of interest of 10 mm^3^ × 10 mm^3^ × 2 mm^3^ was selected, and the parameters such as bone volume/total volume (BV/TV), bone mineral density (BMD), trabecular thickness/number/separation (Tb.Th, Tb.N, Tb.Sp), and structure model index (SMI) were calculated.

### 2.6 Immunohistochemistry staining

The paraffin sections were incubated at 65°C overnight, dewaxed by xylene, and rehydrated, followed by antigen retrieval with 0.1% trypsin for 20 min at 37°C. Subsequently, the sections were incubated with 3% hydrogen peroxide (H_2_O_2_) for 10 min to remove endogenous peroxidase and blocked with Goat serum for 20 min at 37°C. Next, the slides were incubated with primary antibodies, including rabbit anti-Adrb2 (13096-1, 1:300, Proteintech, Wuhan, China), rabbit anti-TH (25859-1-AP, 1:500, Proteintech, Wuhan, China), and rabbit anti-osteocalcin (OCN; 23418-1-AP, 1:300, Proteintech, Wuhan, China) overnight at 4°C. The following day, the sections were treated with horseradish peroxidase-conjugated streptavidin detection system (PV-9001, ZSGB-Bio, Beijing, China) to detect the immunoreactivity. The sections were visualized using 3,3′-diaminobenzidine (ZLI-9018, ZSGB-Bio, Beijing, China), while hematoxylin (ZLI-9610, ZSGB-Bio, Beijing, China) was used for counterstaining. Routine dehydration and mounting were performed. After image capture, the number of positively stained cells was quantified using Image-Pro Plus 6.0 (Media Cybernetics, Inc.) as described previously [21,22]. Then, the histological score was calculated as the average optical density of positively stained cells, which equals the ratio of the overall optical density of the positive cells to the positive area. Positive cells are those with clear brownish-yellow granules in the cytoplasm or nucleus.

### 2.7 Immunofluorescence

The procedure for the first day of the immunofluorescence protocol was the same as that used for immunohistochemistry (described above). The following day, the sections were incubated with goat anti-rabbit IgG-TRITC-conjugated secondary antibody (SA00007-2, 1:500, Proteintech, Wuhan, China) for 1 h at 37°C in the dark. To identify the cell morphology of the nuclei, the sections were counterstained with 4′6-diamidino-2-phenylindole (DAPI; Solarbio, Beijing, China) for 3 min followed by PBS washes.

For double-labeling immunofluorescence, primary antibodies of Adrb2 were used as described earlier. To detect osteoclast cell in the tibial plateau subchondral bone, anti-TRAP antibody (D-3) (1:300, sc-376875, Santa Cruz, Texas, USA) was used as a primary antibody. The mixture of rabbit anti-Adrb2 antibody and mouse anti-TRAP antibody was incubated overnight at 4°C, washed in PBS, and incubated with goat anti-rabbit IgG-TRITC-conjugated secondary antibody (SA00007-2, 1:500, Proteintech, Wuhan, China) and goat anti-mouse IgG-fluorescein isothiocyanate (FITC)-conjugated secondary antibody (SA00003-1, 1:500, Proteintech, Wuhan, China) for 1 h at 37°C in the dark. The sections were then incubated with DAPI and then stimulated under a fluorescent microscope.

All sections were mounted in a fluorescence microscope (DM4B, Leica Microsystems GmbH) for examination. For immunofluorescence analysis, in a place with high light intensity, under a 200× magnification, the optical density value was analyzed under the same area and was equal to the ratio of the overall optical density to the area.

### 2.8 Western blot analysis

From each patient, samples of the subchondral bone MTP and LTP were analyzed by Western blot to determine the protein levels. Briefly, the subchondral bone specimen was defrosted, weighed, and ground in liquid nitrogen, and the tissue was homogenized in cold RIPA lysis buffer (P0013B, Beyotime Biotechnology, Nantong, Jiangsu, China). The total protein sample kit (KGP250, KeyGen Biotech, Nanjing, China) was utilized according to the manufacturer’s instructions, and the protein concentration was determined by the bicinchoninic acid method. An equivalent amount of protein was separated by sodium dodecyl sulfate–polyacrylamide gel electrophoresis (PG112, EpiZyme, Shanghai, China) and transferred to the polyvinylidene fluoride membrane (ISEQ00010, Millipore, Billerica, MA, USA). Then, the membrane was blocked with 5% skim milk for 1 h at room temperature and probed with rabbit polyclonal antibodies raised against Adrb2 (13096-1, 1:500, Proteintech, Wuhan, China), TH (25859-1-AP, 1:500, Proteintech, Wuhan, China), OCN (23418-1-AP, 1:500, Proteintech, Wuhan, China), or GAPDH (10494-1-AP, 1:2,000, Proteintech, Wuhan, China) overnight at 4°C. Subsequently, the membrane was incubated with horseradish peroxidase goat anti-rabbit IgG (ZB-2301, 1:2,000, ZSGB-Bio, Beijing, China) at room temperature for 1 h.

The immunoreactive bands were visualized by electrochemiluminescence on an ALS4000 gel image analysis system (GE Healthcare Life Sciences, Logan, UT, USA). Image J software (NIH, Bethesda, MD, USA) was used to quantify the protein band signal intensity and expressed as arbitrary units (a.u). The optical density was normalized to that of GAPDH to calculate the levels of the target protein.

### 2.9 Reverse transcription-quantitative PCR

The medial and lateral samples of subchondral bone were taken from the tibial plateau of each patient to evaluate the mRNA levels of the target genes. Briefly, the subchondral bone samples were thawed, weighed, and homogenized in liquid nitrogen. Subsequently, total RNA was extracted from the tissues using the RNA pure tissue kit (G3640, Wuhan Servicebio Technology, Wuhan, China), according to the manufacturer’s instructions. Total RNA amount and purity were determined by UV spectrophotometry. The primers for Adrb2, TH, and OCN were designed and synthesized by Servicebio Biotech (Table 1). Total RNA was reverse transcribed into cDNA using the RT First Strand cDNA Synthesis kit (G3331, Wuhan Servicebio Technology, Wuhan, China), according to the manufacturer’s instructions. A 25 µL quantitative polymerase chain reaction (qPCR) kit consisted of cDNA (0.5 µL), SYBR Green qPCR Master Mix (G3320, Wuhan Servicebio Technology, Wuhan, China) (12.5 µL), forward and reverse primers (0.5 µL each), and diethylpyrocarbonate-treated water (11 µL). The amplification was carried out at 50°C (20 min), 95°C (10 min), and 40 cycles at 95°C (15 s) and 60°C (60 s). Then, the mRNA level was calculated using the cycle threshold method (2^−ΔΔCt^) and normalized against that of GAPDH that was used as an internal control.

**Table 1:** Primer sequences used in RT-qPCR

Gene	Accession no.	*T*_m_ (°C)	Product size (bp)	Forward	Reverse
*ADRB2*	NM_000024.6	60	169	GGGTCTTTCAGGAGGCCAAA	ATGCCTAACGTCTTGAGGGC
*TH*	NM_000360.3	60	167	GACCCTGACCTGGACTTGGA	AGCGTGGTGTAGACCTCCTTCC
*OCN*	NM_199173.4	60	110	GGCGCTACCTGTATCAATGG	GTGGTCAGCCAACTCGTCA
*GAPDH*	NM_000360.3	60	168	GGAAGCTTGTCATCAATGGAAATC	TGATGACCCTTTTGGCTCCC

*T*_m_, annealing temperature; Adrb2, β2-adrenergic receptors; TH, tyrosine hydroxylase; OCN, osteocalcin.

### 2.10 Statistical analysis

Statistical analysis was performed using SPSS version 20.0 software (IBM, Chicago, IL, USA). Graphs were drawn using GraphPad Prism version 8.0 software (GraphPad Software Inc., San Diego, USA). The data were presented as mean  ±  SD. The clinical, micro-CT, histology, and immunohistochemistry data were tested for normality using the Shapiro-Wilk Test. Paired sample *t*-test was used to compare the measurements. *P* < 0.05 was considered statistically significant.

## 3 Results

### 3.1 Macroscopic observations

The degeneration of MTP cartilage was more severe than that of LTP cartilage in all specimens. The surface of the MTP cartilage was rough, dull, and grayish-yellow, with extensive softening foci, huge fissures, exposed ivory-like subchondral bone covered by pannus tissue, and multiple osteophytes in the center and edge of the plateau. The surface of the LTP cartilage was flat and locally shiny, with scattered superficial ulcers and softening foci, rare fissure formation, no cartilage loss in the whole layer, no pannus tissue formation, and marginal osteophytes in some specimens (Figure 1b).

### 3.2 Histological assessment

Cartilage damage was investigated by performing Safranin O/fast green staining. The results revealed no notable damage and mild damage in the LTP and severe damage in the MTP. In the MTP, some of the subchondral bone was no longer covered by cartilage, and the subchondral bone was completely exposed. Compared to the MTP, the subchondral trabecular bone of the LTP had thinner and smaller, the chondrocytes of the LTP were relatively normal in size and shape, and the cartilage matrix was stained uniformly. In the MTP sample, the ivory-like subchondral bone, the chondrocytes were abnormal in morphology, swollen, and reduced in number. The cartilage matrix was not stained. Some samples displayed cartilaginous deposits (triangle) among trabecular bones (Figure 1c). Cartilage damage was evaluated using the OARSI score (Table 2). The OARSI scores in the MTP were higher than that in the LTP. The analysis showed that there were statistically significant differences in OARSI score among groups (*P* < 0.0001) (Figure 1c). The TAC thickness of the MTP subchondral bone was significantly lower than that of the LTP, while BA/TA and SCP thickness were significantly higher than those of the LTP subchondral bone (*P* < 0.0001) (Table 2).

**Table 2:** Structural parameters of the cartilage and subchondral bone of the medial tibial plateau and the lateral tibial plateau of the knee joint were evaluated by Safranin O/Fast Green staining (mean ± SD)

Groups	OARSI score	BA/TA (%)	TAC (µm)	SCP (µm)
LTP	9.97 ± 3.19	22.42 ± 3.33	2277.69 ± 236.72	212.72 ± 33.81
MTP	21.73 ± 2.56	49.46 ± 3.06	148.38 ± 26.55	1268.78 ± 171.04
Statistic	*P* < 0.0001	*P* < 0.0001	*P* < 0.0001	*P* < 0.0001

OARSI, osteoarthritis research society international; TAC, total articular cartilage; SCP, subchondral bone plate; BA/TA, trabecular bone area/total area; MTP, medial tibial plateau; LTP, medial tibial plateau.

### 3.3 Micro-CT evaluation

**Figure 2:**
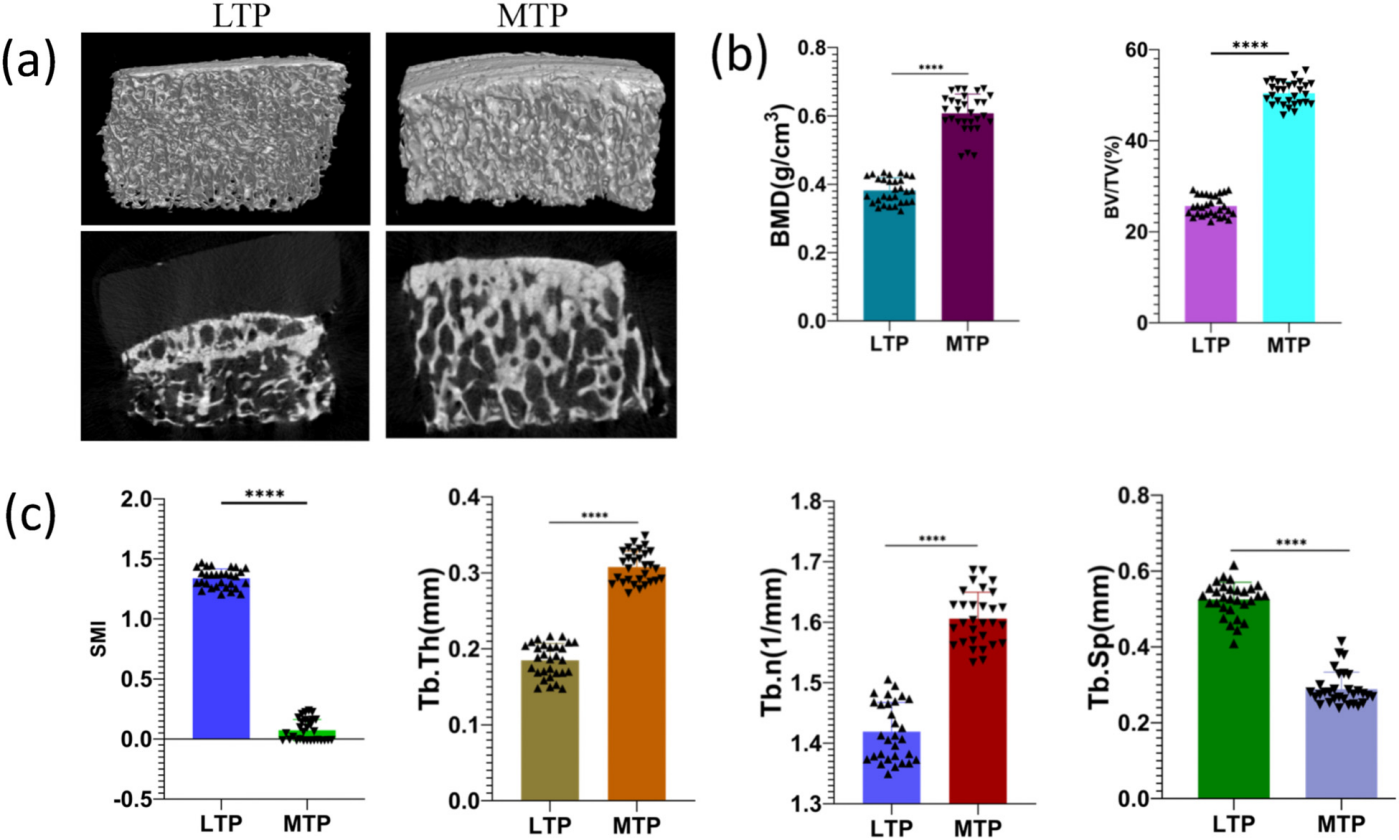
Micro-CT evaluation of the MTP and LTP of subchondral bone. (a) 2D and 3D micro-CT images. (b and c) Quantitative micro-CT analysis of tibial subchondral bone of bone volume fraction (BV/TV), bone mineral density (BMD), trabecular thickness/number/separation (Tb.Th, Tb.N, Tb.Sp), and structure model index (SMI). The difference between the MTP and LTP groups was statistically significant. ^****^ *P* < 0.0001.

The micro-CT 2D images revealed that the MTP subchondral bone had increased bone mass and reduced porosity compared to the LTP subchondral bone. The micro-CT 3D images show significant thickening of the SCP in the MTP compared to the LTP (Figure 2a). The measurement of bone structural parameters showed that BV/TV, Tb.N, and Tb.Th of the medial subchondral bone were significantly increased compared to those of the LTP subchondral bone, while SMI and Tb.SP were significantly decreased (*P* < 0.0001; Figure 2b and c).

### 3.4 TRAP staining

**Figure 3:**
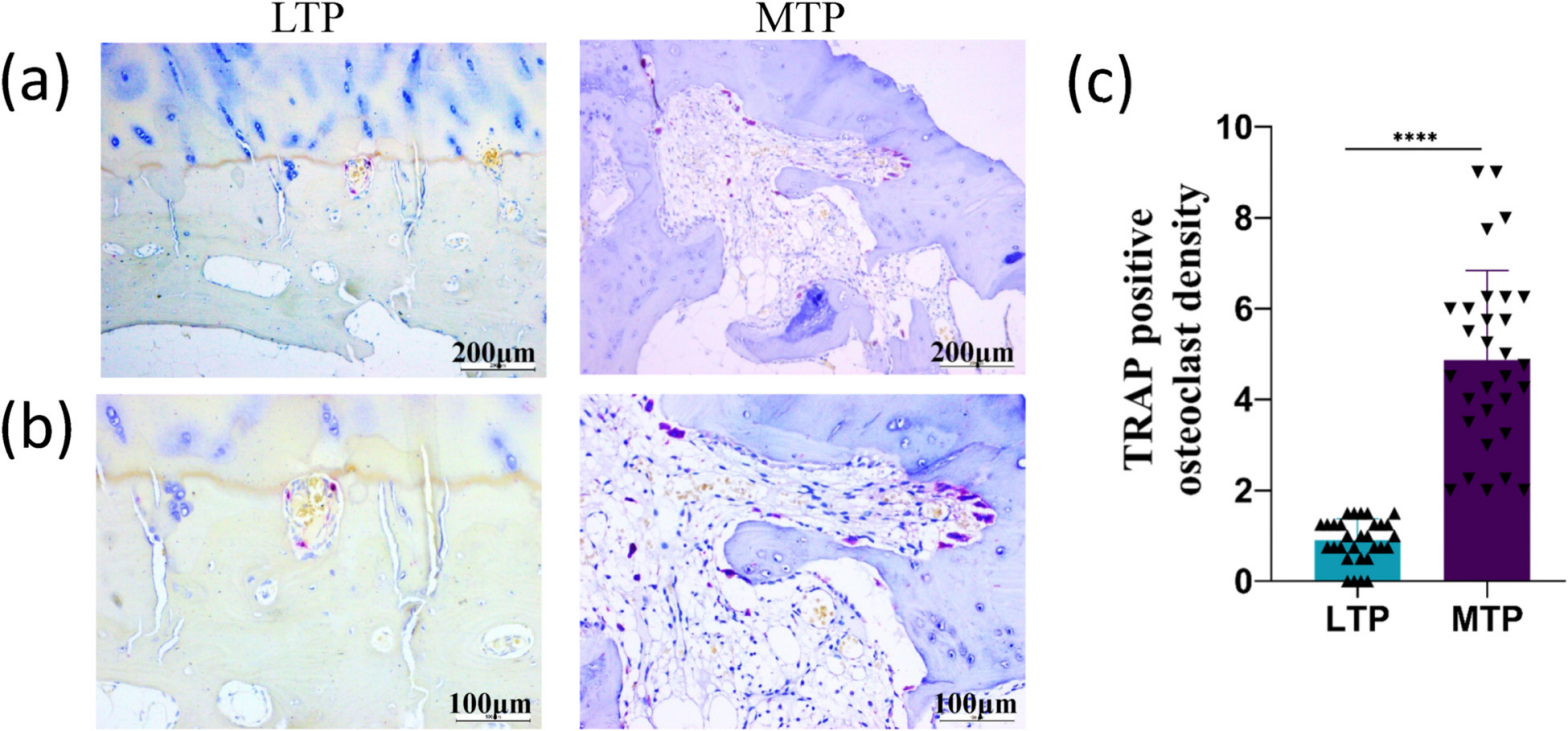
Comparison of TRAP-positive osteoclasts in the subchondral bone of LTP and MTP. (a) A large number of TRAP-positive cells were distributed in the MTP subchondral bone. In the LTP subchondral bone, TRAP-positive cells were rarely distributed. (b) TRAP-positive cells mainly distributed in the MTP subchondral bone absorption pit region. TRAP-positive cells mainly distributed at the junction of cartilage and subchondral bone in the LTP. (c) TRAP-positive osteoclasts were significantly higher with MTP compared to LTP. (a) Magnification ×100, scale bar 200 µm; (b) magnification × 200, scale bar 100 µm; ^****^ *P* < 0.0001.

TRAP-positive multinucleated osteoclasts are observed at the bone surface of the subchondral bone (Figure 3). In the MTP, subchondral bone TRAP-positive cells were widely distributed (Figure 3a), mainly distributed in the subchondral bone resorption pit region (Figure 3b). In the lateral tibia platform, subchondral bone TRAP-positive cells were rarely distributed (Figure 3a), mainly distributed in the junction between cartilage and subchondral bone (Figure 3b). The density of osteoclasts in the subchondral bone in the MTP was significantly higher than in the LTP (*P* < 0.0001; Figure 3c).

### 3.5 Adrb2, OCN, and TH levels detected by immunohistochemistry

**Figure 4:**
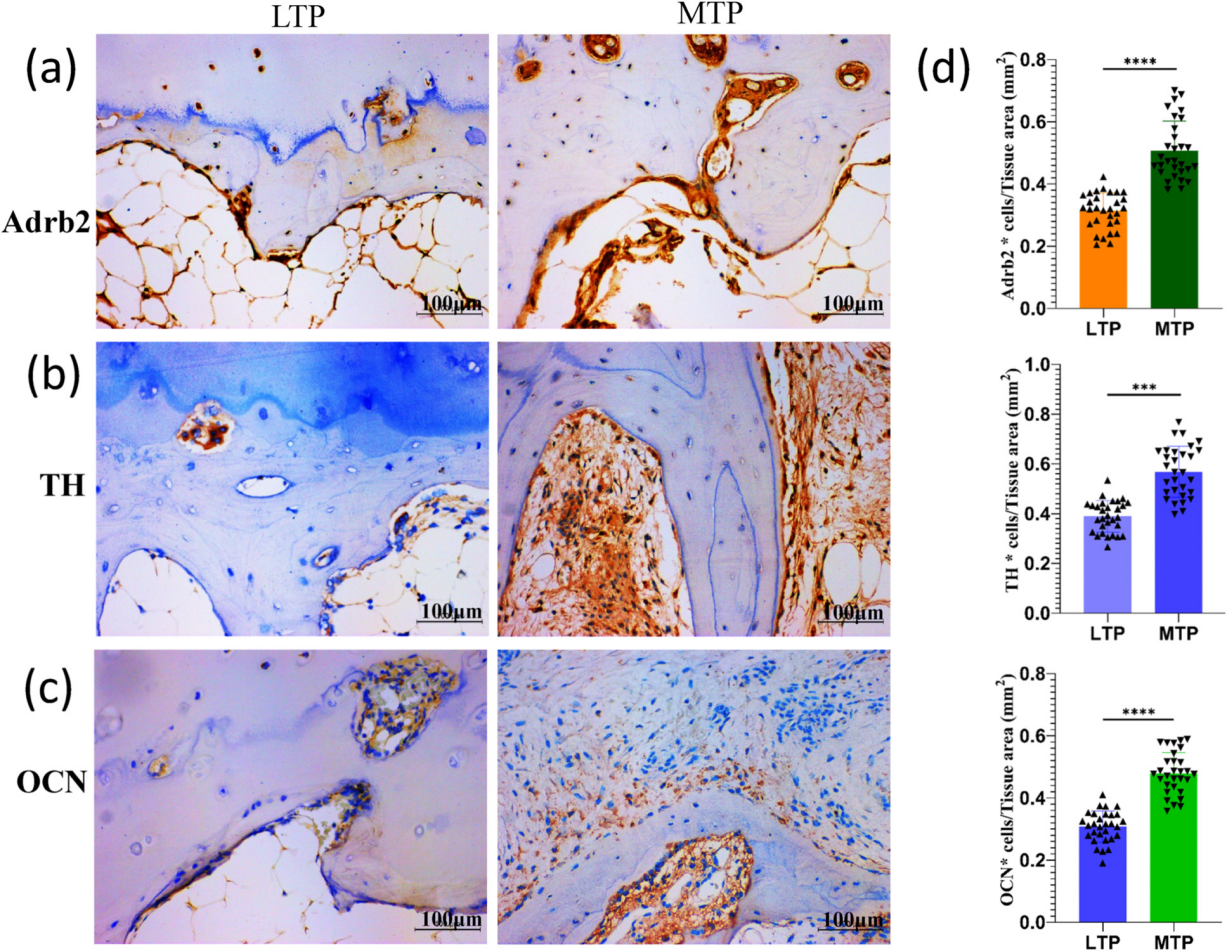
Immunohistochemistry analysis of Adrb2, TH and OCN expression in subchondral bone of LTP and MTP (a–c) Representative images of Adrb2, TH, and OCN expression in the subchondral bone of LTP and MTP. (d) Bar graphs show semiquantitative evaluation of Adrb2, TH, and OCN immunohistochemistry. Magnification ×200, scale bar 100 µm; ^***^ *P* < 0.001, ^****^ *P* < 0.0001.

Adrb2 is expressed in both osteoblasts and osteoclasts, but mainly in the cell membrane. The MTP subchondral bone Adrb2-positive cells are multinucleated clusters, primarily distributed in the trabecular bone edge and the subchondral bone resorption pits. The LTP subchondral bone Adrb2-positive cells constitute the distribution channel in bone cartilage and subchondral bone edge (Figure 4a). The MTP subchondral bone Adrb2-positive cell density was significantly higher than that of the LTP subchondral bone (*P* < 0.0001; Figure 4d). The TH-positive cells were also partially expressed in cartilage, but mainly distributed in the subchondral bone resorption pits and bone marrow cavities (Figure 4b). The MTP subchondral bone density was significantly higher than TH-positive cells in the LTP subchondral bone (*P* < 0.001; Figure 4d). OCN is mainly expressed in the cell membrane and intercellular space of osteoblasts, with a scattered gritty distribution (Figure 4c). The LTP subchondral bone OCN-positive cell density was significantly lower than that of the MTP subchondral bone (*P* < 0.0001; Figure 4d).

### 3.6 Adrb2, OCN, and TH levels detected by immunofluorescence

**Figure 5:**
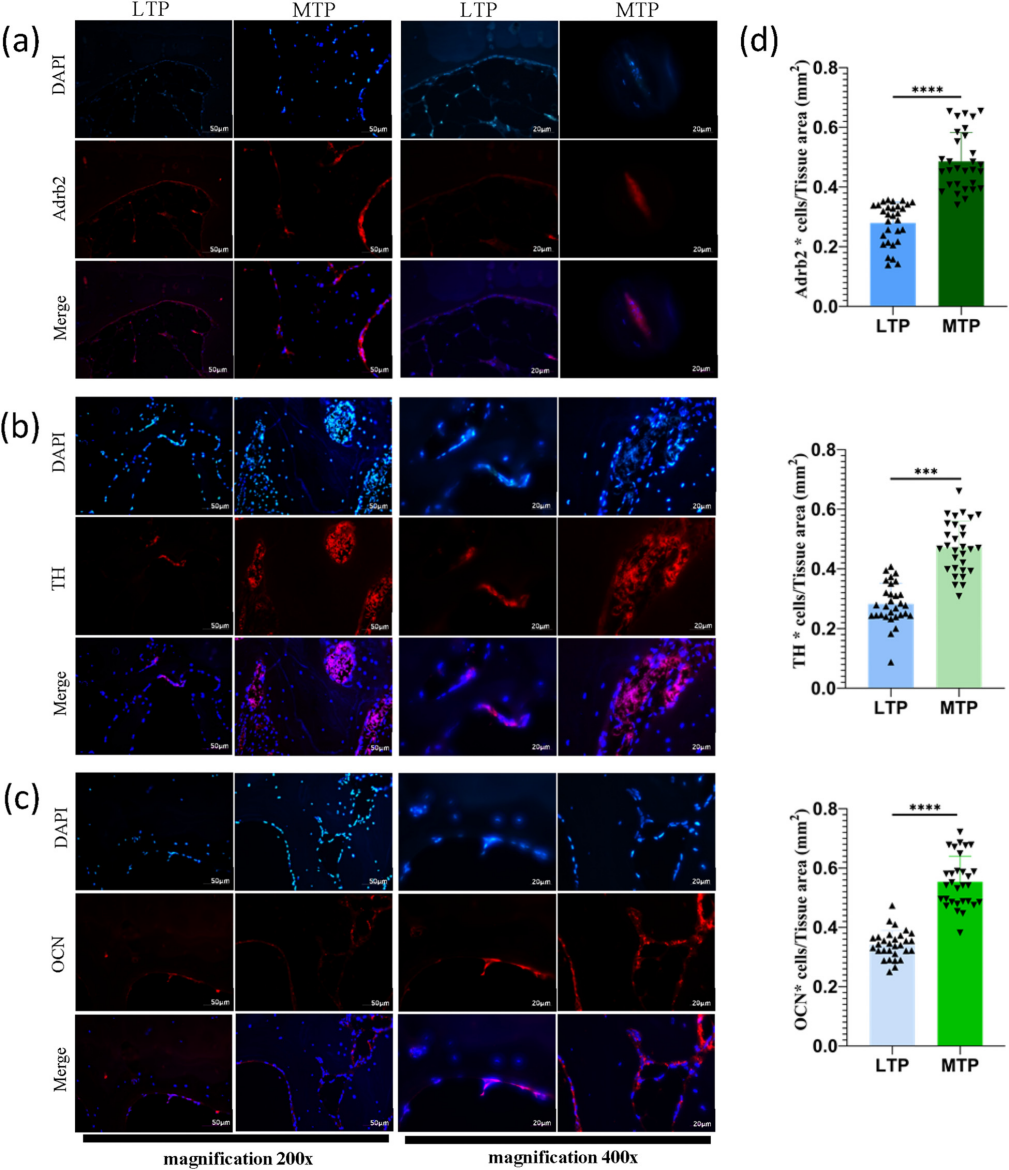
Immunofluorescence analysis of Adrb2, TH, and OCN expression in subchondral bone of LTP and MTP. (a) In the subchondral bone of MTP, Adrb2 (red) is mainly expressed on the cell membrane of multinucleated cells. In the subchondral bone of LTP, Adrb2 was mainly expressed on the membrane of multinucleated cells at the edge of the subchondral bone. (b) TH (red) is mainly distributed in the edge of the subchondral bone and the bone marrow cavity. (c) OCN (red) is distributed on the edge of the subchondral bone and is mainly expressed in the cell membrane and intercellular space of osteoblasts, presenting granular distribution. Nuclei were stained with DAPI (blue). (d) Bar graphs show semiquantitative evaluation of Adrb2, TH, and OCN immunofluorescence. Magnification × 200, scale bar 50 µm; magnification ×400, scale bar 20 µm; ^***^ *P* < 0.001, ^****^ *P* < 0.0001.

Immunofluorescence staining was performed using TRITC, which is excited at 550 nm and emits red light, and then images of the samples were captured using a 40X fluorescence microscope. The results of Adrb2 (Figure 5a), TH (Figure 5b), and OCN (Figure 5c) expression were observed and statistically analyzed (Figure 5d). The differences in Adrb2, TH, and OCN expression were statistically significant between the MTP subchondral bone and LTP subchondral bone by analyzing the mean optical density (*P* < 0.0001, *P* < 0.001, and *P* < 0.0001, respectively).

### 3.7 Evaluation of Adrb2 and TRAP co-expression by double-labeling immunofluorescence

**Figure 6:**
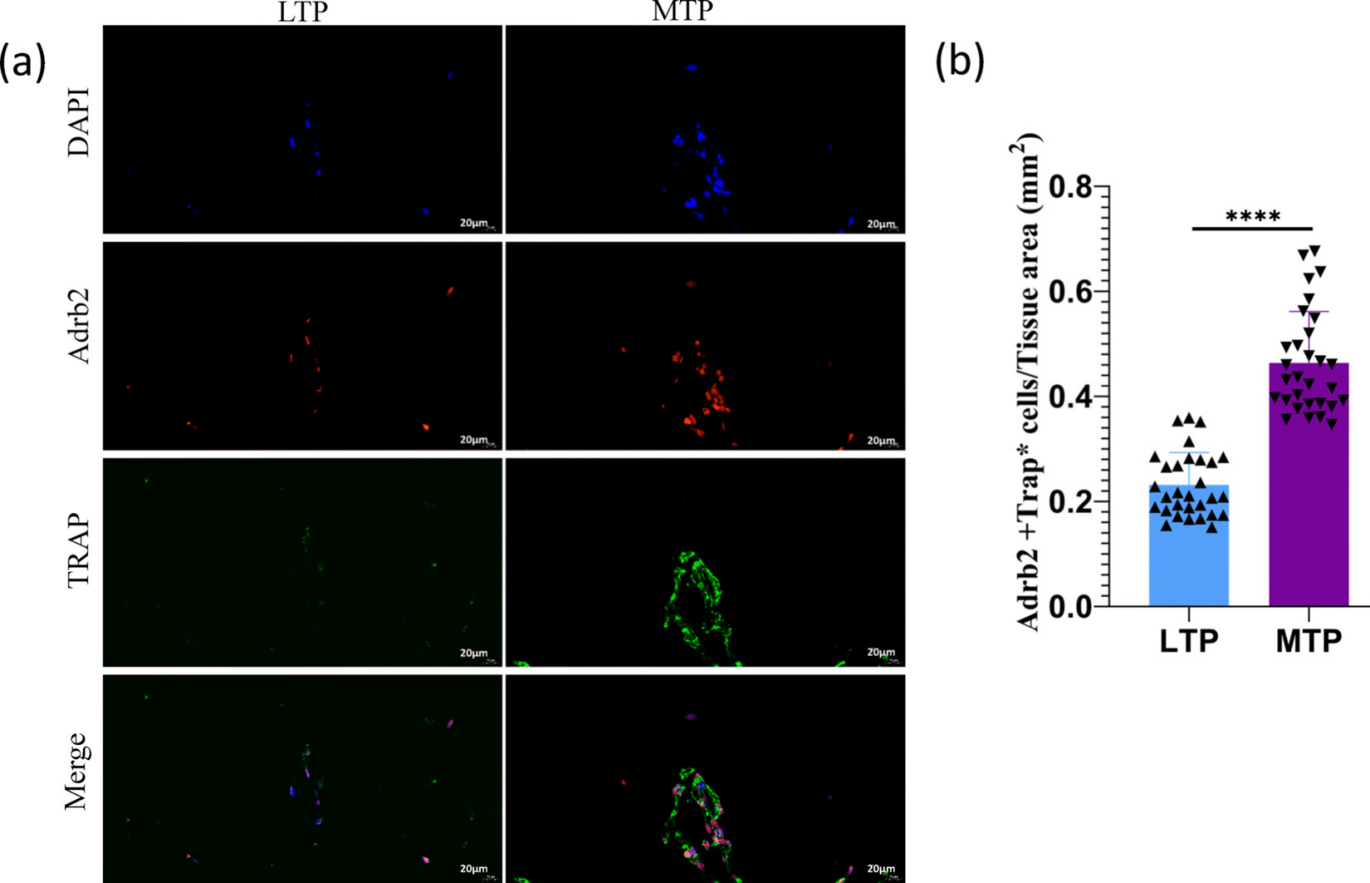
Immunofluorescence double-labeling staining analysis of Adrb2 and TRAP co-expression in subchondral bone of LTP and MTP. (a) Distribution of Adrb2 and TRAP in the subchondral bone of LTP and MTP. Double immunofluorescence of Adrb2 (red)/TRAP (green) labeled multinucleated cells. Nuclei were stained with DAPI (blue). (b) Bar graphs show semiquantitative evaluation of Adrb2 and TRAP co-expression immunofluorescence. Magnification ×400, scale bar 20 µm; ^****^ *P* < 0.0001.

To further observe Adrb2 and TRAP co-expression levels in osteoclasts from the subchondral bone, double-labeling immunofluorescence staining was used. In the double-labeling immunofluorescence staining, TRAP expression was performed using FITC, which is excited at 500 nm and emits green light, while Adrb2 expression was the same as before, and then images of the samples were captured using a 40X fluorescence microscope. Adrb2 and TRAP were detected in both MTP and LTP subchondral bones. Double-labeling immunofluorescence showed that Adrb2 was present in the majority of TRAP-positive multinuclear cells of the MTP subchondral bone (Figure 6a). By analyzing the mean optical density, the difference in Adrb2 and TRAP co-expression was statistically significant between MTP and LTP subchondral bone (*P* < 0.0001; Figure 6b).

### 3.8 Adrb2, OCN, and TH protein levels in subchondral bone determined by Western blot analysis

**Figure 7:**
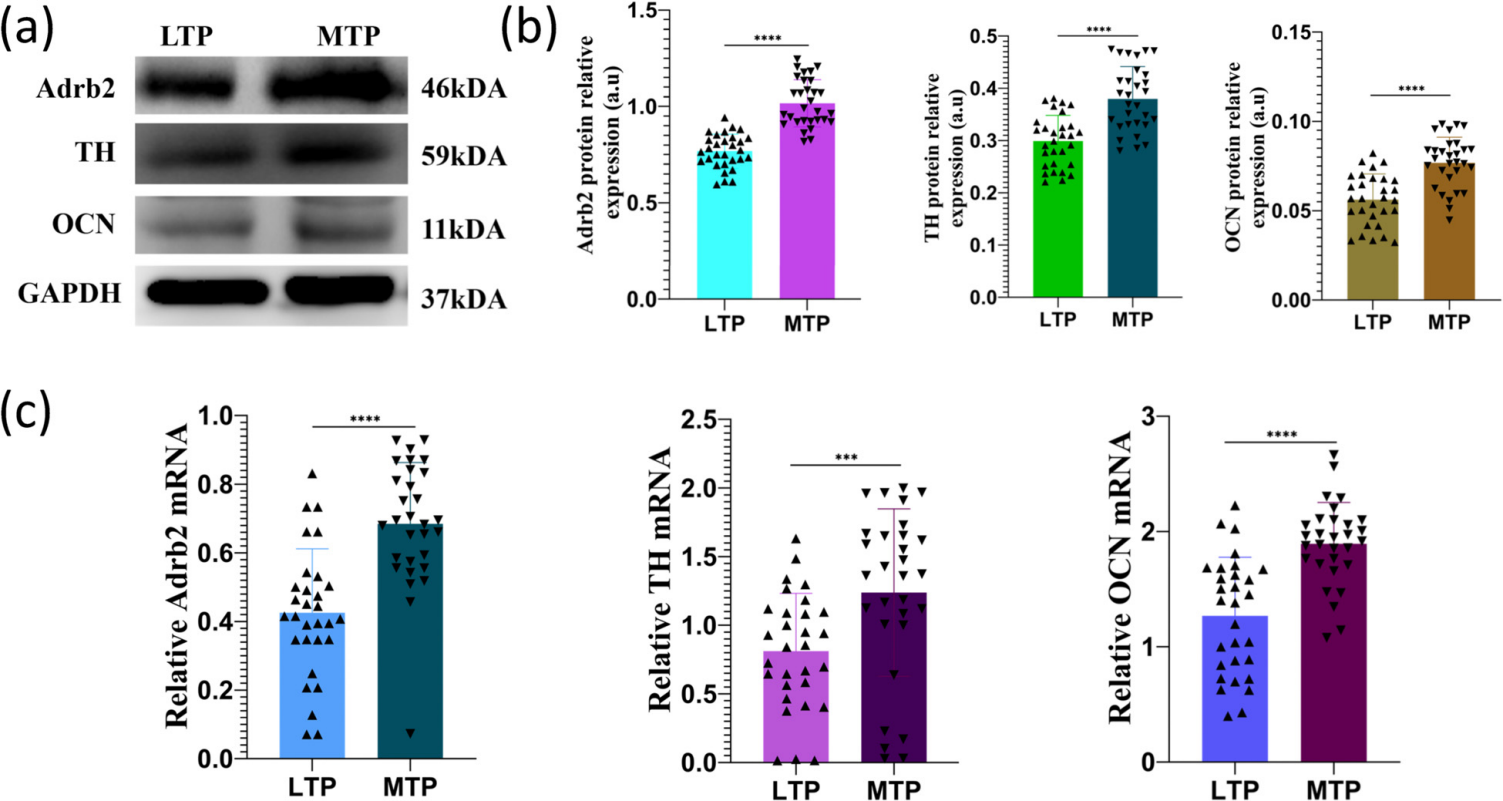
The expression levels of Adrb2, TH, and OCN in the subchondral bone of MTP and LTP. (a) Representative Western blots showing the expression of Adrb2, TH, and OCN. (b) Relative expression of Adrb2, TH, and OCN protein levels in the subchondral bone of MTP and LTP of knee osteoarthritis. GAPDH was used for normalization. (c) Semiquantitative reverse transcription PCR analysis of Adrb2, TH, and OCN in MTP and LTP subchondral bone in knee osteoarthritis. ^***^ *P* < 0.001, ^****^ *P* < 0.0001.

Western blot results showed the protein levels of Adrb2, OCN, and TH in subchondral bone of MTP and LTP by the gray scale of the bands (Figure 7a). The densities of the bands were numerically quantified and compared. Compared to the values obtained from the LTP subchondral bone samples, the protein levels of Adrb2, OCN, and TH of the MTP subchondral bone samples increased (*P* < 0.0001; Figure 7b).

### 3.9 Adrb2, OCN, and TH mRNA levels in subchondral bone

Compared to the expression obtained from the LTP subchondral bone samples, the mRNA levels of Adrb2, OCN, and TH of the MTP subchondral bone samples increased (*P* < 0.0001, *P* < 0.001, and *P* < 0.0001, respectively; Figure 7c).

## 4 Discussion

In this study, we investigated changes in subchondral bone remodeling, microstructure, and their association with cartilage degradation in KO varus deformity patients. We found that MTP had abnormal bone remodeling and microstructural deterioration and cartilage severe damage. In addition, the level of OCN in the MTP subchondral bone was higher than that in the LTP, which was consistent with the positive correlation between increased subchondral osteogenesis and the progression of OA reported previously [23]. The results of micro-CT also showed significant differences in the bone structure between medial and lateral subchondral bone, which was consistent with the results of the study by Finnilä et al. [24]. These findings indicated that the severe the OA, the obvious the degree of subchondral bone hyperplasia and osteosclerosis.

In this study, we selected the tibial plateau with varus deformity of KO and found that the mechanical stresses of the MTP were higher than that of the LTP, and the lower limb mechanical stresses and mechanical axis of the lower limb were correlated with the local BMD [25]. The adduction moment of the knee is a major determinant of medial to lateral load distribution and also the cause of the biomechanical abnormality of KO in the medial compartment [11,26]. Intriguingly, lower limb dynamic load and tibial KO disease severity have been reported, and adduction torque increase might affect the medial tibial platform structure of OA and cause varus deformity [27]. Varus alignment increases the risk of progression of medial ventricular OA in KO [28]. The knee adductive moment reflects the dynamic load in the medial joint compartment and predicts the progression of radiographic OA [27,29]. Therefore, for patients with varus deformity of KO, medial OA is severe than lateral OA because the mechanical loading on the MTP is greater than that on LTP.

The SNS distributed in the periosteum, bone trabecula, bone marrow, and subchondral bone strongly affects bone remodeling [5,30]. In this study, we detected TH through testing the subchondral bone in MTP and LTP and illustrated that the sympathetic nerve is involved in the subchondral bone of bone remodeling. The previous animal model studies have shown that SNS suppresses bone formation by activating Adrb2 on osteoblasts, directly or indirectly accelerates osteoclast generation, and promotes bone loss [7,9]. The present study demonstrated Adrb2 in the human KO subchondral bone, and as far as we know, it was also the first report. To avoid differences in the results due to individual factors (such as hormone levels, weight, age, and occupation), we took the same knee joint on the medial and lateral sides of the tibial plateau for a paired comparison study. Interestingly, the expression of Adrb2 in the MTP subchondral bone with high bone mass, and obviously, osteosclerosis was higher than that in LTP and was mainly distributed in the subchondral bone absorption socket.

It has been reported that Adrb2 was expressed in both osteoblasts and osteoclasts [31]. To determine whether Adrb2 expression originated in osteoclasts, immunofluorescence and TRAP staining were performed. Immunofluorescence showed that Adrb2 was mainly expressed in multinucleated osteoclasts. TRAP staining showed that the number of osteoclasts in MTP subchondral bone was significantly higher than that of LTP, and they were mainly distributed in the subchondral bone absorption pit, which was similar to the distribution of Adrb2. Our study revealed that TRAP and Adrb2 were co-expressed in multinucleated cells by immunofluorescence double-label detection, and the expression level of MTP was higher than that of LTP. In this study, we did not use *in vitro* cell cultures for the detection of Adrb2 because Adrb2 serves as hormone receptors, and the properties of these cells change when they are removed from their environment. Thus, we inferred that Adrb2 on MTP with high mechanical stress is mainly expressed on osteoclasts. A comparative study on the medial sclerotic area and the lateral nonsclerotic area of the tibial plateau in patients with KO found that the number and the activity of osteoclasts in the sclerotic area were significantly higher than that in the nonsclerotic area [32]. The increased activity of osteoclasts in the sclerotic area of the bone indicated that the activity of bone formation and resorption were increased in human KO, but no coupling was observed.

Sympathetic mediator catechins induce differentiation of osteoclast precursors and stimulate osteoclast activity by binding to Adrb2 [33]. Since Adrb2 is a hormone receptor, the subchondral bone of the MTP and LTP of the knee of the same patient was used to conduct a paired experiment, suggesting that there were differences in topical catecholamines in the subchondral bone between MTP and LTP. This suggests that mechanical stress is the main cause of upregulation of Adrb2 in the subchondral bone, inducing osteoclast generation and participating in subchondral bone remodeling. Wnt signal promotes the proliferation and differentiation of osteoblasts [34]. Mature osteoclasts can reduce the Wnt signaling pathway inhibitor osteosclerosis protein expression and through the secretion of Wnt10b and BMP6 promoting osteoclast areas of osteoblast differentiation and bone formation [35]. Our research team has previously reported that the severity of KO is negatively correlated with the expression of sclerostin in the subchondral bone [36]. Recent studies have shown that osteoclasts downregulate the expression of sclerostin in the trabecular bone [37], which can explain the fact that the sclerotic MTP has high osteogenic activity and high osteoclast density in this study.

Pain is the main cause of disability in KO patients [38], and subchondral bone is the main cause of KO pain [39,40]. In overweight and obese KO patients, knee pressure and pain can be reduced by weight loss [41]. Similarly, high tibial osteotomy improved the mechanical axis of the lower limbs and relieved the load of the medial compartment of the knee, thus alleviating pain [42,43]. These studies suggest a positive correlation between intraarticular mechanical stresses and pain [44]. In studies on symptomatic KO, it was found that the increased nerve growth factor and osteoclast density in osteochondral channels seem to be key factors leading to KO bone pain [45]. In this study, we selected patients who underwent total knee arthroplasty due to medial knee pain. Therefore, we speculated that the MTP with high mechanical stress might elevate the number of osteoclasts through the high expression of β2-adrenergic receptor in the subchondral bone, leading to joint pain. In another study, β-adrenergic receptor inhibitors reduced the need for opioids in patients with joint pain and OA [46], which indirectly supports our view. However, a further study of the role of mechanical stress through the activation of subchondral bone osteoblasts by Adrb2 to regulate osteoblasts in OA, animal model experiments is still needed.

The current research related to the pathogenesis of KO of the most common animal models includes destabilization of medial meniscus and anterior cruciate ligament transection, etc., which are posttraumatic OA models and cannot objectively reflect abnormal subchondral bone remodeling of OA caused by mechanical stresses. The alveolar bone has a strong remodeling ability after mechanical stimulation, such as bite force, which is considered an ideal model for studying the bone mechanical response. In the OTM, mechanically modulating Adrb2 induces and promotes osteoclast generation and regulates alveolar bone remodeling [7]. In a model of OA of the temporomandibular joint, increased osteoclast activity by intraperitoneal injection of Adrb2 agonist caused bone loss following increased mechanical stresses [9]. Adrb2 is a G protein-coupled receptor [47], a signal converter that converts extracellular signals into intracellular signals; plays a key role in bone development, remodeling, and diseases; and is the primary drug target of human diseases [48]. It provides a new target for treating OA and alleviating symptoms.

In summary, we compared the MTP subchondral bone with the LTP subchondral bone in patients with varus knee deformity using a number of experimental methods. The bone structure of the MTP subchondral bone was significantly different from that of the LTP subchondral bone, showing increased bone mass and obvious sclerosis. However, the density of osteoclasts and OCN in the MTP subchondral bone were higher than that in the LTP subchondral bone, indicating that there was no coupling between bone formation and resorption. Furthermore, it is reported for the first time that the expression of Adrb2 in the MTP subchondral bone with high mechanical stresses is higher than that in the LTP subchondral bone, indicating that the signal transduction of Adrb2 plays an important role in mechanical stress-induced subchondral bone remodeling. However, the mechanism of Adrb2 in the whole pathogenesis of OA needs to be further studied, which may provide a new target for the treatment of OA and the reduction of symptoms.

## List of abbreviations

Adrb2: activates the β2-adrenergic receptor
BCA: bicinchoninic acid
BMD: bone mineral density
BV: bone volume
DAB: diaminobenzidine
EDTA: ethylene diamine tetra acetic acid
GAPDH: glyceraldehyde phosphate dehydrogenase
HTO: High tibial osteotomy
KO: knee osteoarthritis
LTP: lateral tibial plateau
MTP: medial tibial plateau
NE: neurotransmitter norepinephrine
OA: osteoarthritis
OCN: osteocalcin
OTM: orthodontic tooth movement
PBS: phosphate-buffered saline
SCP: subchondral bone plate
SD: standard deviation
SEM: standard error of the mean
SMI: structure model index
SNS: sympathetic nervous system
TAC: total articular cartilage
TH: tyrosine hydroxylase
TMJ: temporomandibular joint
TRAP: tartrate-resistant acid phosphatase

## References

[1] Hunter J, Bierma-zeinstra S. Osteoarthritis. Lancet. 2019;393(10182):1745–59. 10.1016/S0140-6736(19)30417-9 31034380

[2] Burr DB, Gallant MA. Bone remodelling in osteoarthritis. Nat Rev Rheumatol. 2012;8(11):665–73. 10.1038/nrrheum.2012.130 22868925

[3] Hill EL, Elde R. Distribution of CGRP-, VIP-, D beta H-, SP-, and NPY-immunoreactive nerves in the periosteum of the rat. Cell Tissue Res. 1991;264(3):469–80. 10.1007/BF00319037 1714353

[4] Qiao Y, Wang Y, Zhou Y, Jiang F, Huang T, Chen L, et al. The role of nervous system in adaptive response of bone to mechanical loading. J Cell Physiol. 2019;234(6):7771–80. 10.1002/jcp.27683 30414185

[5] Elefteriou F, Campbell P, Ma Y. Control of bone remodeling by the peripheral sympathetic nervous system. Calcif Tissue Int. 2014;94(1):140–51. 10.1007/s00223-013-9752-4 PMC3883940 23765388

[6] Courties A, Sellam J, Berenbaum F. Role of the autonomic nervous system in osteoarthritis. Best Pract Res Clin Rheumatol. 2017;31(5):661–75. 10.1016/j.berh.2018.04.001 30509412

[7] Cao H, Kou X, Yang R, Liu D, Wang X, Song Y, et al. Force-induced Adrb2 in periodontal ligament cells promotes tooth movement. J Dental Res. 2014;93(11):1163–9. 10.1177/0022034514551769 PMC4293769 25252876

[8] Pongratz G, Straub RH. Role of peripheral nerve fibres in acute and chronic inflammation in arthritis. Nat Rev Rheumatol. 2013;9(2):117–26. 10.1038/nrrheum.2012.181 23147892

[9] Jiao K, Niu LN, Li QH, Ren GT, Zhao CM, Liu YD, et al. β2-Adrenergic signal transduction plays a detrimental role in subchondral bone loss of temporomandibular joint in osteoarthritis. Sci Rep. 2015;5:12593. 10.1038/srep12593 PMC4518212 26219508

[10] Dayal N, Chang A, Dunlop D, Hayes K, Chang R, Cahue S, et al. The natural history of anteroposterior laxity and its role in knee osteoarthritis progression. Arthritis Rheumatism. 2005;52(8):2343–9. 10.1002/art.21277 16052589

[11] Schipplein OD, Andriacchi TP. Interaction between active and passive knee stabilizers during level walking. J Orthopaedic Res Off Publ Orthopaedic Res Soc. 1991;9(1):113–9. 10.1002/jor.1100090114 1984041

[12] Hügle T, Geurts J. What drives osteoarthritis? -synovial versus subchondral bone pathology. Rheumatol (Oxford, Engl). 2017;56(9):1461–71. 10.1093/rheumatology/kew389 28003493

[13] Palmer JS, Jones LD, Monk AP, Nevitt M, Lynch J, Beard DJ, et al. Varus alignment of the proximal tibia is associated with structural progression in early to moderate varus osteoarthritis of the knee. Knee Surgery Sports Traumatol Arthroscopy Off J ESSKA. 2020;28(10):3279–86. 10.1007/s00167-019-05840-5 PMC7511471 31965215

[14] Hunt MA, Charlton JM, Esculier JF. Osteoarthritis year in review 2019: mechanics. Osteoarthr Cartil. 2020;28(3):267–74. 10.1016/j.joca.2019.12.003 31877382

[15] Altman R, Asch E, Bloch D, Bole G, Borenstein D, Brandt K, et al. Development of criteria for the classification and reporting of osteoarthritis. Classification of osteoarthritis of the knee. Diagnostic and therapeutic criteria committee of the american rheumatism association. Arthritis Rheumatism. 1986;29(8):1039–49. 10.1002/art.1780290816 3741515

[16] Kellgren JH, Lawrence JS. Radiological assessment of osteo-arthrosis. Ann Rheumatic Dis. 1957;16(4):494–502. 10.1136/ard.16.4.494 PMC1006995 13498604

[17] Grogan SP, Barbero A, Winkelmann V, Rieser F, Fitzsimmons JS, O'driscoll S, et al. Visual histological grading system for the evaluation of in vitro-generated neocartilage. Tissue Eng. 2006;12(8):2141–9. 10.1089/ten.2006.12.2141 16968155

[18] Pritzker KP, Gay S, Jimenez SA, Ostergaard K, Pelletier JP, Revell PA, et al. Osteoarthritis cartilage histopathology: grading and staging. Osteoarthr Cartil. 2006;14(1):13–29. 10.1016/j.joca.2005.07.014 16242352

[19] Nwosu LN, Allen M, Wyatt L, Huebner JL, Chapman V, Walsh DA, et al. Pain prediction by serum biomarkers of bone turnover in people with knee osteoarthritis: an observational study of TRAcP5b and cathepsin K in OA. Osteoarthr Cartil. 2017;25(6):858–65. 10.1016/j.joca.2017.01.002 28087412

[20] Wen CY, Chen Y, Tang HL, Yan CH, Lu WW, Chiu KY. Bone loss at subchondral plate in knee osteoarthritis patients with hypertension and type 2 diabetes mellitus. Osteoarthr Cartil. 2013;21(11):1716–23. 10.1016/j.joca.2013.06.027 23831668

[21] Zhen G, Wen C, Jia X, Li Y, Crane JL, Mears SC, et al. Inhibition of TGF-β signaling in mesenchymal stem cells of subchondral bone attenuates osteoarthritis. Nat Med. 2013;19(6):704–12. 10.1038/nm.3143 PMC3676689 23685840

[22] Wang T, Wen CY, Yan CH, Lu WW, Chiu KY. Spatial and temporal changes of subchondral bone proceed to microscopic articular cartilage degeneration in guinea pigs with spontaneous osteoarthritis. Osteoarthr Cartil. 2013;21(4):574–81. 10.1016/j.joca.2013.01.002 23313833

[23] Nevitt MC, Zhang Y, Javaid MK, Neogi T, Curtis JR, Niu J, et al. High systemic bone mineral density increases the risk of incident knee OA and joint space narrowing, but not radiographic progression of existing knee OA: the MOST study. Ann Rheumatic Dis. 2010;69(1):163–8. 10.1136/ard.2008.099531 PMC2935624 19147619

[24] Finnilä MAJ, Thevenot J, Aho OM, Tiitu V, Rautiainen J, Kauppinen S, et al. Association between subchondral bone structure and osteoarthritis histopathological grade. J Orthopaedic Res. 2017;35(4):785–92. 10.1002/jor.23312 PMC5412847 27227565

[25] Wada M, Maezawa Y, Baba H, Shimada S, Sasaki S, Nose Y. Relationships among bone mineral densities, static alignment and dynamic load in patients with medial compartment knee osteoarthritis. Rheumatol (Oxford, Engl). 2001;40(5):499–505. 10.1093/rheumatology/40.5.499 11371657

[26] Andriacchi TP. Dynamics of knee malalignment. Orthopedic Clin North Am. 1994;25(3):395–403. 8028883

[27] Miyazaki T, Wada M, Kawahara H, Sato M, Baba H, Shimada S. Dynamic load at baseline can predict radiographic disease progression in medial compartment knee osteoarthritis. Ann Rheumatic Dis. 2002;61(7):617–22. 10.1136/ard.61.7.617 PMC1754164 12079903

[28] Sharma L, Song J, Felson DT, Cahue S, Shamiyeh E, Dunlop DD. The role of knee alignment in disease progression and functional decline in knee osteoarthritis. JAMA. 2001;286(2):188–95. 10.1001/jama.286.2.188 11448282

[29] Garcia SA, Vakula MN, Holmes SC, Pamukoff DN. The influence of body mass index and sex on frontal and sagittal plane knee mechanics during walking in young adults. Gait Posture. 2021;83:217–22. 10.1016/j.gaitpost.2020.10.010 33171375

[30] Sseur R, Sabatier JP, Potrel-Burgot C, Lecoq B, Creveuil C, Marcelli C. Sympathetic nervous system as transmitter of mechanical loading in bone. Jt Bone Spine. 2003;70(6):515–9. 10.1016/j.jbspin.2003.07.006 14756119

[31] Togari A. Adrenergic regulation of bone metabolism: possible involvement of sympathetic innervation of osteoblastic and osteoclastic cells. Microscopy Res Tech. 2002;58(2):77–84. 10.1002/jemt.10121 12203706

[32] Geurts J, Patel A, Hirschmann MT, Pagenstert GI, Müller-Gerbl M, Valderrabano V, et al. Elevated marrow inflammatory cells and osteoclasts in subchondral osteosclerosis in human knee osteoarthritis. J Orthopaedic Res Off Publ Orthopaedic Res Soc. 2016;34(2):262–9. 10.1002/jor.23009 26250062

[33] Frediani U, Becherini L, Lasagni L, Tanini A, Brandi ML. Catecholamines modulate growth and differentiation of human preosteoclastic cells. Osteoporos Int J Established Result Cooperation Eur Found Osteoporos Natl Osteoporos Found USA. 1996;6(1):14–21. 10.1007/BF01626532 8845594

[34] Khosla S, Westendorf JJ, Oursler MJ. Building bone to reverse osteoporosis and repair fractures. J Clin Investigation. 2008;118(2):421–8. 10.1172/JCI33612 PMC2214701 18246192

[35] Pederson L, Ruan M, Westendorf JJ, Khosla S, Oursler MJ. Regulation of bone formation by osteoclasts involves Wnt/BMP signaling and the chemokine sphingosine-1-phosphate. Proc Natl Acad Sci U S A. 2008;105(52):20764–9. 10.1073/pnas.0805133106 PMC2603259 19075223

[36] Wu L, Guo H, Sun K, Zhao X, Ma T, Jin Q. Sclerostin expression in the subchondral bone of patients with knee osteoarthritis. Int J Mol Med. 2016;38(5):1395–402. 10.3892/ijmm.2016.2741 PMC5065295 27665782

[37] Koide M, Yamashita T, Murakami K, Uehara S, Nakamura K, Nakamura M, et al. Sclerostin expression in trabecular bone is downregulated by osteoclasts. Sci Rep. 2020;10(1):13751. 10.1038/s41598-020-70817-1 PMC7426814 32792620

[38] Neogi T. The epidemiology and impact of pain in osteoarthritis. Osteoarthr Cartil. 2013;21(9):1145–53. 10.1016/j.joca.2013.03.018 PMC3753584 23973124

[39] Aso K, Shahtaheri SM, McWilliams DF, Walsh DA. Association of subchondral bone marrow lesion localization with weight-bearing pain in people with knee osteoarthritis: data from the Osteoarthritis Initiative. Arthritis Res Ther. 2021;23(1):35. 10.1186/s13075-021-02422-0 PMC7816469 33468243

[40] Driban JB, Price L, Lo GH, Pang J, Hunter DJ, Miller E, et al. Evaluation of bone marrow lesion volume as a knee osteoarthritis biomarker--longitudinal relationships with pain and structural changes: data from the Osteoarthritis Initiative. Arthritis Res Ther. 2013;15(5):R112. 10.1186/ar4292 PMC3978948 24020939

[41] Messier SP, Mihalko SL, Legault C, Miller GD, Nicklas BJ, DeVita P, et al. Effects of intensive diet and exercise on knee joint loads, inflammation, and clinical outcomes among overweight and obese adults with knee osteoarthritis: the IDEA randomized clinical trial. JAMA. 2013;310(12):1263–73. 10.1001/jama.2013.277669 PMC4450354 24065013

[42] Liu X, Chen Z, Gao Y, Zhang J, Jin Z. High Tibial Osteotomy: Review of Techniques and Biomechanics. J Healthc Eng. 2019;2019:8363128. 10.1155/2019/8363128 PMC6525872 31191853

[43] He M, Zhong X, Li Z, Shen K, Zeng W. Progress in the treatment of knee osteoarthritis with high tibial osteotomy: a systematic review. Syst Rev. 2021;10(1):56. 10.1186/s13643-021-01601-z PMC7883424 33583421

[44] Christensen P, Henriksen M, Bartels EM, Leeds AR, Meinert Larsen T, Gudbergsen H, et al. Long-term weight-loss maintenance in obese patients with knee osteoarthritis: a randomized trial. Am J Clin Nutr. 2017;106(3):755–63. 10.3945/ajcn.117.158543 28747328

[45] Aso K, Shahtaheri SM, Hill R, Wilson D, McWilliams DF, Walsh DA. Associations of symptomatic knee osteoarthritis with histopathologic features in subchondral bone. Arthritis Rheumatol (Hoboken, NJ). 2019;71(6):916–24. 10.1002/art.40820 30663865

[46] Nakafero G, Grainge M, Valdes A, Townsend N, Mallen C, Zhang W, et al. Do β-adrenoreceptor blocking drugs associate with reduced risk of symptomatic osteoarthritis and total joint replacement in the general population? A primary care-based, prospective cohort study using the Clinical Practice Research Datalink. BMJ Open. 2019;9(8):e032050. 10.1136/bmjopen-2019-032050 PMC6688671 31375622

[47] Luo J, Sun P, Siwko S, Liu M, Xiao J. The role of GPCRs in bone diseases and dysfunctions. Bone Res. 2019;7:19. 10.1038/s41413-019-0059-6 PMC6804689 31646011

[48] Sriram K, Insel PA. G protein-coupled receptors as targets for approved drugs: how many targets and how many drugs? Mol Pharmacol. 2018;93(4):251–8. 10.1124/mol.117.111062 PMC5820538 29298813

